# Severe COVID-19 in inflammatory bowel disease patients in a population-based setting

**DOI:** 10.1371/journal.pone.0258271

**Published:** 2021-10-05

**Authors:** Rob H. Creemers, Ashkan Rezazadeh Ardabili, Daisy M. Jonkers, Mathie P. G. Leers, Mariëlle J. Romberg-Camps, Marie J. Pierik, Ad A. van Bodegraven

**Affiliations:** 1 Department of Gastroenterology, Geriatrics, Internal and Intensive Care Medicine (Co-MIK), Zuyderland Medical Centre, Heerlen-Sittard-Geleen, The Netherlands; 2 Department of Internal Medicine, Division of Gastroenterology and Hepatology, Maastricht University Medical Centre+, Maastricht, The Netherlands; 3 School for Nutrition and Translational Research in Metabolism (NUTRIM), Maastricht University Medical Centre+, Maastricht, The Netherlands; 4 Department of Clinical Chemistry and Haematology, Zuyderland Medical Centre, Heerlen-Sittard-Geleen, The Netherlands; Center for Primary Care and Public Health, SWITZERLAND

## Abstract

**Objective:**

Data on the course of severe COVID-19 in inflammatory bowel disease (IBD) patients remains limited. We aimed to determine the incidence rate and clinical course of severe COVID-19 in the heavily affected South-Limburg region in the Netherlands.

**Methods:**

All COVID-19 patients admitted to the only two hospitals covering the whole South-Limburg region between February 27, 2020 and January 4, 2021 were included. Incidence rates for hospitalization due to COVID-19 were determined for the IBD (n = 4980) and general population (n = 597,184) in South-Limburg.

**Results:**

During a follow-up of 4254 and 510,120 person-years, 20 IBD patients (0.40%; 11 ulcerative colitis (UC), 9 Crohn’s disease (CD)) and 1425 (0.24%) patients from the general population were hospitalized due to proven COVID-19 corresponding to an incidence rate of 4.7 (95% Confidence interval (CI) 3.0–7.1) and 2.8 (95% CI 2.6–2.9) per 1000 patient years, respectively (Incidence rate ratio: 1.68, 95% CI 1.08–2.62, p = 0.019). Median age (IBD: 63.0 (IQR 58.0–75.8) years vs. general population: 72.0 (IQR 62.0–80.0) years, p = 0.10) and mean BMI (IBD: 24.4 (SD 3.3) kg/m^2^ vs. general population 24.1 (SD 4.9) kg/m^2^, p = 0.79) at admission were comparable in both populations. As for course of severe COVID-19, similar rates of ICU admission (IBD: 12.5% vs. general population: 15.7%, p = 1.00), mechanical ventilation (6.3% vs. 11.2%, p = 1.00) and death were observed (6.3% vs. 21.8%, p = 0.22).

**Conclusion:**

We found a statistically significant higher rate of hospitalization due to COVID-19 in IBD patients in a population-based setting in a heavily impacted Dutch region. This finding reflects previous research that showed IBD patients using systemic medication were at an increased risk of serious infection. However, although at an increased risk of hospitalization, clinical course of severe COVID-19 was comparable to hospitalized patients without IBD.

## Introduction

Millions of people, including patients with Inflammatory Bowel Diseases (IBD), have been affected by severe acute respiratory syndrome coronavirus 2 (SARS-CoV-2). The broad use of immunosuppressants and biologicals in IBD patients increases susceptibility to opportunistic or severe infections [1–3]. Coronavirus disease 2019 (COVID-19) is an infectious disease caused by SARS-CoV-2 [4]. Data on incidence and clinical course of severe COVID-19 compared to a general population remains relatively sparse [5–12]. Especially data from population-based settings in heavily affected regions are underrepresented.

In a recent Swedish population-based study, IBD patients were at an increased risk of hospitalization for COVID-19 compared to the general population, even after adjusting for comorbidities [13]. Remarkably, the subsequent risk for intensive care unit (ICU) admission or death was comparable with a low absolute risk of severe COVID-19 in IBD patients [13]. To guide vaccination strategies against SARS-CoV-2, it is crucial to exactly delineate populations at risk for a severe course of COVID-19.

South-Limburg is a border region in the Southeast of the Netherlands with 597,184 inhabitants enclosed by Germany and Belgium [14]. All regional inpatient care is provided by two hospitals: Zuyderland Medical Centre, a large general district hospital, and Maastricht University Medical Centre+. During the COVID-19 pandemic, the second highest COVID-19 mortality rate (144.7 per 100,000) in the Netherlands (96.0 national average) has been reported for this region [15].

This report aimed to expand the current knowledge on severe COVID-19 in the IBD population by calculating the incidence rate and examining the clinical course of severe COVID-19 compared to the regional population in a population-based setting in South-Limburg.

## Materials and methods

### Patient cohorts and follow-up

The IBD-cohort comprised all patients in the South-Limburg region with an IBD diagnosis at February 27^th^ 2020 in either one of the only two hospitals covering this region. Due to lockdown and travel limitations all regional COVID-19 patients were treated in these hospitals. The total IBD population in South-Limburg is currently estimated at 4980 patients.

As control population, the total number of inhabitants in the South-Limburg region on February 27^th^, 2020 (n = 597,184) were included. Population data were obtained from the Dutch Central Bureau of Statistics (CBS) [14].

Both populations were observed from February 27^th^, 2020, date of first confirmed COVID-19 diagnosis in the Netherlands, until January 4^th^, 2021, the start date of COVID-19 vaccinations in the Netherlands.

### Outcome and definitions

This study had two main outcomes: (1) incidence rate of severe COVID-19 was calculated for both IBD patients and the regional population. All IBD patients from the South Limburg region who were admitted for COVID-19-related complaints were included. For the regional population, data on COVID-19 hospitalization were obtained from The Dutch National Institute for Public Health and Environment (RIVM) which publishes daily updated metadata.

(2) To compare clinical course of severe COVID-19 in IBD patients with comparators, baseline characteristics and data on the course of severe COVID-19 were collected for all severe COVID-19 cases in Zuyderland Medical Centre. For this outcome, severe COVID-19 was defined as a confirmed COVID-19 diagnosis (*i*.*e*., COVID-19 associated symptoms and either a positive SARS-CoV-2 polymerase chain reaction or CT-CORADS score (≥4)) requiring hospitalization, and/or resulting in ICU admission or death. All patients admitted to Zuyderland Medical Centre were prospectively registered in the Zuyderland COVID-19 Registry (ELVIS). After inclusion, patients with IBD in their medical history were identified based on the ICD-10 codes.

### Ethics

This study was approved by the Ethics Committee of Zuyderland Medical Centre (Z2020171). All COVID-19 patients admitted to Zuyderland MC were registered in the ZuydErLand COVID-19 regiStry (ELVIS). All patients in the ELVIS registry received written information about the registry as well as an opt-out form in case they did not want to participate. None of the patients included in this study objected to participation. This opt-out mechanism was approved by the Ethics Committee of Zuyderland Medical Centre.

### Statistics

Incidence rates were calculated as the ratio between COVID-19 hospitalizations (events) and patient-years of follow-up (time). Difference between incidence rates was assessed by calculating the incidence rate ratio using the ‘Exact Poisson Method’ [16]. Baseline characteristics are presented as means with standard deviations (SD) or medians with interquartile range (IQR) for normal or non-normal distributed numerical variables respectively, and as number of patients with corresponding percentage for categorical variables. Independent samples t-test or Mann-Whitney U test was used for comparison of numerical variables and Chi-square test for categorical variables. Statistical analyses were conducted using SPSS (Version 26.0). A two-sided P value ≤0.05 was considered statistically significant.

## Results

### Incidence rate of severe COVID-19

During a follow-up of 4254 person-years, 20 IBD patients (0.40%; 11UC, 9CD) were hospitalized in the South-Limburg region due to COVID-19 corresponding to an incidence rate of 4.7 (95% Confidence interval (CI) 3.0–7.1) per 1000 person-years. During the same period, in the general population 1425 patients (0.24%) were admitted to the hospital with COVID-19 during 510,120 person-years yielding an incidence rate of 2.8 (95% CI 2.6–2.9) per 1000 person-years and translating to an incidence rate ratio of 1.68 (95% CI 1.08–2.62, *p* = 0.019) for IBD versus the general population (Table 1).

**Table 1:** Risk of severe COVID–19 (*i*.*e*. hospitalization) in IBD patients and the general population in South–Limburg between February 27, 2020 and January 4, 2021.

	N (%)		N events (%)		Time at risk (person years, PY)		Incidence rate per 1000 PY (95% CI)		Incidence rate ratio (95% CI, *p* value)	
Outcome	IBD	General population	IBD	General population	IBD	General population	IBD	General population	IBD	General population
Main outcome										
Severe COVID-19	4980	597,184	20 (0.40%)	1425 (0.24%)	4254	510,120	4.7 (3.0–7.1)	2.8 (2.6–2.9)	1.68 (1.08–2.62;***p* = 0.019**)	REF

N, number of patients; N events, number of severe COVID–19 events; IBD, inflammatory bowel disease; PY, person years; CI, confidence interval.

### Outcome of severe COVID-19

Out of 870 patients hospitalized for severe COVID-19 in the Zuyderland MC, 16 patients had IBD (10 UC, 6 CD). Baseline characteristics for the study population are presented in Table 2. Median age and BMI at admission were comparable between the IBD population and comparators. A lower, but statistically non-significant, burden of comorbidities (Charlson comorbidity index (CCI) ≥4 37.5% vs. 56.7%) was registered in the IBD population.

**Table 2:** Baseline and severe COVID–19 course characteristics of the IBD population and regional matched time frame comparators.

Baseline characteristics	IBD (n = 16)	Comparators (n = 854)	p-value
Females, n (%)	6 (37.5)	317 (37.1)	0.98
Males, n (%)	10 (62.5)	537 (62.9)	
Age at COVID-19 diagnosis, median (IQR)	63.0 (58.0–75.8)	72.0 (62.0–80.0)	0.10
Age categories			0.51
18–49, n (%)	1 (6.3)	58 (6.8)	
50–69, n (%)	8 (50.0)	316 (37.0)	
>70, n (%)	7 (43.8)	480 (56.2)	
Charlson Comorbidity Index			0.17
CCI = 0, n (%)	1 (6.3)	53 (6.2)	
CCI = 1, n (%)	3 (18.8)	79 (9.3)	
CCI = 2, n (%)	1 (6.3)	105 (12.3)	
CCI = 3, n (%)	5 (31.1)	133 (15.6)	
CCI> = 4, n (%)	6 (37.5)	484 (56.7)	
BMI, mean (SD)^a^	24.4 (3.3)	24.1 (4.9)	0.79
IBD disease entity			-
Ulcerative Colitis, n (%)	10 (62.5)	-	
Crohns Disease, n (%)	6 (37.5)	-	
Medication IBD, n (%)^b^	10 (62.5)		-
Mesalazine, n (%)	5 (50.0)	-	
Thiopurine, n (%)	2 (20.0)	-	
(Topical) corticosteroids, n (%)	4 (40.0)	-	
Anti-TNF, n (%)	0 (0.0)	-	
Combination therapy, n (%)^c^	2 (20.0)	-	
IBD PGA			-
Remission	12 (75.0)	-	
Mild	2 (12.5)	-	
Moderate	2 (12.5)	-	
Severe	0 (0.0)	-	
**Severe COVID-19 characteristics**	**IBD (n = 16)**	**Comparators (n = 854)**	**p-value**
Symptom onset, median days (IQR)	7.0 (7.0–9.8)	7.0 (3.0–10.0)	0.36
Symptoms			
Respiratory, n (%)^d^	13 (86.7)	733 (87.9)	1.00
Gastrointestinal, n (%)^e^	9 (69.2)	347 (62.5)	0.78
Fever, n (%)^f^	11 (100.0)	430 (78.5)	0.13
Duration of hospital stay, median days (IQR)	8.5 (3.0–16.3)	6.0 (4.0–12.0)	0.51
Medical Intervention			
Antiviral, n (%)^g^	4 (25.0)	214 (25.1)	1.00
Chloroquine, n (%)	6 (37.5)	272 (31.9)	0.79
Corticosteroids, n (%)^h^	4 (25.0)	233 (27.4)	1.00
Tocilizumab, n (%)^i^	2 (12.5)	55 (6.5)	0.28
Antifungal, n (%)^j^	0 (0.0)	16 (1.9)	1.00
Oxygen support, n (%)	11 (68.8)	709 (83.0)	0.17
NIV, n (%)^k^	2 (20.0)	118 (21.5)	1.00
Mechanical ventilation, n (%)	1 (6.3)	96 (11.2)	1.00
ICU admission, n (%)	2 (12.5)	134 (15.7)	1.00
ICU duration, median days (IQR)	-	9.50 (4.00–21.00)	-
Range	6–15	1–102	-
Death, n (%)	1 (6.3)	186 (21.8)	0.22

IBD, Inflammatory bowel disease; CCI, Charlson Comorbidity Index; BMI, Body Mass Index; PGA, Physician global assessment; NIV, Non–invasive ventilation; ICU, intensive care unit; *n*, number of patients; SD, standard deviation; IQR, interquartile range.

^a^ No data available on BMI in *n* = 1 and *n* = 62 IBD patients and comparators, respectively

^b^ Medication categories are not mutually exclusive

^c^ One patient used topical beclomethasone and mesalazine, and one patient used methotrexate and sulfasalazine

^d^ No data available on respiratory symptoms in *n* = 1 and *n* = 20 IBD patients and comparators, respectively

^e^ No data available on gastrointestinal symptoms in *n* = 3 and *n* = 299 IBD patients and comparators, respectively

^f^ No data available on fever in *n* = 5 and *n* = 306 IBD patients and comparators, respectively

^g^ No data available on antiviral therapy in *n = 1* comparator

^h^ No data available on corticosteroid therapy in *n = 3* comparators

^i^ No data available on tocilizumab therapy in *n = 4* comparators

^j^ No data available on antifungal therapy *n = 5* comparators

^k^ No data available on NIV in *n* = 6 and *n* = 306 IBD patients and comparators, respectively.

As for clinical course of severe COVID-19, rates of oxygen support, ICU admission, and mechanical ventilation did not differ between IBD patients versus matched time frame comparators without IBD. Furthermore, no statistically significant difference in death rate date was observed (Table 2).

## Discussion

In the present study, we aimed to investigate the incidence rate and clinical outcomes of severe COVID-19 for the IBD population. We observed a statistically significant increase in hospitalization rates due to COVID-19 in a population of 4980 IBD patients when compared to the general population in South-Limburg. This finding reflects previous research in a large population based nationwide cohort study that showed that IBD patients using systemic medication were at an increased risk of serious infections [1]. It is important to note that although we demonstrated an increased risk of hospitalization, the risk of a serious course of COVID-19 was not different in the IBD population compared to the matched time frame comparator population without IBD.

Epidemiological data and disease outcome data on severe COVID-19 in IBD patients from the Netherlands and Denmark have previously been reported [7, 8]. In these studies, SARS-CoV-2 testing data from the general population were compared with data on symptomatic COVID-19 in IBD patients, likely to overrepresent the number of cases in the general population. In another recent population-based study from Sweden, it was assessed whether IBD patients were at an increased risk of severe COVID-19 [13]. In the latter study, patients were identified at hospital admission, and IBD patients were matched with general population controls, therefore enabling more precise risk estimations. Reported incidence rates were 5.4 (95%CI 4.6–6.2), and 3.4 (95%CI 3.1–3.7) per 1000 patient years for COVID-19 hospital admissions in the IBD population and matched controls, respectively. In the present study, similar incidence rates for COVID-19 hospitalization (IBD: 4.7, 95%CI 3.0–7.1 vs. Comparators: 2.8, 95%CI 2.6–2.9) were observed.

Regarding the course of severe COVID-19, baseline patient characteristics of both groups did not differ. Interestingly, BMI, a well-established risk factor for worse disease outcome, was not different between the two groups and equal to the Dutch average [17, 18]. On post-hoc analysis, BMI of patients requiring ICU admission was significantly higher compared to non-ICU patients in both groups (mean BMI 25.8 (SD: 4.6) vs mean BMI 23.9 (SD: 4.8), p<0.001), confirming the importance of BMI as a risk factor for severe COVID-19. Additionally, the clinical course of severe COVID-19 was comparable between IBD patients and comparators, as reported before in different settings [19–22].

Interestingly, none of the IBD patients with severe COVID-19 were on biologicals when in fact a substantial proportion of IBD patients in our population currently use biologicals, in particular anti-tumour necrosis factor (TNF) agents such as infliximab and adalimumab. Since TNF is recognized as an important component of the cytokine response during the inflammatory phase, also known as the cytokine release syndrome (CRS), several studies have described the rationale for anti-TNF therapy in COVID-19 [23, 24]. Moreover, accumulating observational clinical data in IBD and other immune mediated inflammatory diseases (IMIDs) have highlighted the potential benefit of anti-TNF therapies as treatment for COVID-19 and have also led to initiation of various clinical trials that are currently investigating use of anti-TNF in COVID-19 [25]. All in all, although cases were limited, the observation of no anti-TNF users among the IBD patients with severe COVID-19 in the current study further substantiates the potential protective role of anti-TNF agents in the pathophysiology of COVID-19.

Among the strengths of this study is the strict definition of severe COVID-19 applied to both the IBD population as well as the matched time frame comparators. Furthermore, the prospective ELVIS registry enabled detailed phenotyping of baseline and COVID-19 characteristics in both IBD patients and comparators, thus providing us with means to find signals that may be missed in larger retrospective cohorts based on selected cases. As therapeutic focus shifts from reducing symptoms of SARS-CoV-2 infection to preventing severe infection through vaccination, the presented data may be of help to identify most benefitting populations since IBD and treatment of it do not seem to worsen clinical course of severe COVID-19. Nevertheless, we recognize several limitations in this study, of which most important the limited number of severe COVID-19 cases. The limited number of cases prevented adjustment of incidence rates for demographic and clinical characteristics and sub-analyses to identify risk factors for severe COVID-19. Therefore, the statistical comparisons should be interpreted with care.

In conclusion, we found a statistically significant higher rate of hospitalization due to COVID-19 in IBD patients in a population-based setting in a heavily impacted Dutch region. This finding reflects previous population-based reports in which was shown that IBD patients using systemic medication are at an increased risk of serious infection, as contrasted with case-based analyses in larger series in which IBD did not seem to be a risk factor for contracting SARS-CoV-2 infection. However, despite increased rates of hospitalization, clinical course of COVID-19 was comparable to hospitalized patients without IBD.

## Abbreviations

CD: Crohn’s disease
COVID-19: Coronavirus disease 2019
CT-CORADS: CT coronavirus disease 2019 reporting and data system
IBD: Inflammatory bowel disease
SARS-CoV-2: Severe acute respiratory syndrome coronavirus 2
UC: Ulcerative colitis
